# 异基因造血干细胞移植治疗骨髓增生异常综合征伴原始细胞增多282例生存分析及WHO 2022分类不同亚型的生存比较

**DOI:** 10.3760/cma.j.cn121090-20231109-00258

**Published:** 2024-05

**Authors:** 慧 王, 润芝 马, 爱明 庞, 栋林 杨, 欣 陈, 荣莉 张, 嘉璘 魏, 巧玲 马, 卫华 翟, 祎 何, 尔烈 姜, 明哲 韩, 四洲 冯

**Affiliations:** 1 中国医学科学院血液病医院（中国医学科学院血液学研究所），北京协和医学院，血液与健康全国重点实验室，国家血液系统疾病临床医学研究中心，细胞生态海河实验室，天津 300020 State Key Laboratory of Experimental Hematology, National Clinical Research Center for Blood Diseases, Haihe Laboratory of Cell Ecosystem, Institute of Hematology & Blood Diseases Hospital, Chinese Academy of Medical Sciences & Peking Union Medical College, Tianjin 300020, China; 2 天津医学健康研究院，天津 301600 Tianjin Institutes of Health Science, Tianjin 301600, China; 3 青岛大学附属烟台毓璜顶医院，烟台 264000 The Affiliated Yantai Yuhuangding Hospital of Qingdao University, Yantai 264000, China

**Keywords:** 骨髓增生异常综合征, 骨髓增生异常肿瘤, 异基因造血干细胞移植, 预后, Myelodysplastic syndrome, Myelodysplastic neoplasm, Allogeneic hematopoietic stem cell transplantation, Prognosis

## Abstract

**目的:**

评估异基因造血干细胞移植（allo-HSCT）治疗骨髓增生异常综合征伴原始细胞增多（MDS-EB）的疗效和预后影响因素，比较WHO2022分类不同亚型患者的预后。

**方法:**

纳入2006年10月至2022年12月在中国医学科学院血液病医院接受allo-HSCT的282例MDS-EB患者，按照WHO 2022诊断标准重新分类为骨髓增生异常肿瘤伴原始细胞增多1型/2型（MDS-IB1/IB2）（222例）、MDS伴纤维化（MDS-f）（41例）和伴双等位基因TP53突变的MDS（MDS-biTP53）（19例）三组，对其临床资料进行回顾性分析。

**结果:**

①282例患者中位年龄46（15～66）岁，男191例，女91例，MDS-EB1 118例（42％），MDS-EB2 164例（58％）。②282例MDS-EB患者中256例（90.8％）移植后获得造血重建，原发植入功能不良11例（3.9％），继发植入功能不良15例（5.3％）。移植后100 d急性移植物抗宿主病（GVHD）累积发生率为（42.6±3.0）％，Ⅱ～Ⅳ度急性GVHD累积发生率为（33.0±2.8）％；移植后1年慢性GVHD累积发生率为（31.0±2.9）％。移植后128例（45.4％）患者发生巨细胞病毒（CMV）感染，63例（22.3％）患者发生菌血症，35例（12.4％）患者发生肺部真菌感染，17例（6.0％）患者发生EB病毒感染。③移植后中位随访时间为22.1（19.2～24.7）个月，3年总生存（OS）率、无病生存（DFS）率分别为71.9％（95％*CI* 65.7％～78.6％）、63.6％（95％*CI* 57.2％～70.7％），3年非复发死亡率（NRM）为17.9％（95％*CI* 13.9％～22.9％），3年累积复发率（CIR）为9.8％（95％*CI* 6.7％～13.7％）。影响移植后OS的独立危险因素包括单体核型（MK）（*P*＝0.004，*HR*＝3.26，95％*CI* 1.46～7.29）、造血干细胞移植合并症指数（HCI-CI）≥3分（*P*<0.001，*HR*＝2.86，95％*CI* 1.72～4.75）、发生Ⅱ～Ⅳ度肠道急性GVHD（*P*<0.001，*HR*＝5.94，95％*CI* 3.50～10.10）。④MDS-IB1/IB2组移植后3年OS率、DFS率均优于MDS-biTP53组［OS：72.0％（95％*CI* 63.4％～80.7％）对46.4％（95％*CI* 26.9％～80.1％），*P*＝0.020；DFS：67.4％（95％*CI* 60.3％～75.3％）对39.7％（95％*CI* 22.3％～70.8％），*P*＝0.015］，3年CIR低于MDS-biTP53组［7.3％（95％*CI* 4.3％～11.4％）对26.9％（95％*CI* 9.2％～48.5％），*P*＝0.004）］。MDS-IB1/IB2组、MDS-f组、MDS-biTP53组移植后3年NRM分别为16.7％（95％*CI* 12.1％～22.1％）、20.5％（95％*CI* 9.4％～34.6％）、26.3％（95％*CI* 9.1％～47.5％）（*P*＝0.690）。

**结论:**

allo-HSCT是MDS-EB的有效治疗手段，单体核型、HCI-CI、Ⅱ～Ⅳ度肠道急性GVHD是影响患者OS的独立危险因素。WHO 2022分类有助于区分不同亚组患者allo-HSCT后疗效，allo-HSCT能够改善MDS-f患者的不良预后，但MDS-biTP53患者移植后复发风险较高。

## Abstract (en)

**Objective:**

To evaluate the efficacy and prognostic factors of allogeneic hematopoietic stem cell transplantation（allo-HSCT）in patients with myelodysplastic syndrome accompanied by myelodysplasia（MDS-EB）and to compare the prognosis of different subtypes of patients classified by World Health Organization（WHO）2022.

**Methods:**

A total of 282 patients with MDS-EB who underwent allo-HSCT at the Hematology Hospital of the Chinese Academy of Medical Sciences from October 2006 to December 2022 were included in the study. The WHO 2022 diagnostic criteria reclassified MDS into three groups: myelodysplastic tumors with type 1/2 of primitive cell proliferation（MDS-IB1/IB2, 222 cases）, MDS with fibrosis（MDS-f, 41 cases）, and MDS with biallelic TP53 mutation（MDS-biTP53, 19 cases）. Their clinical data were retrospectively analyzed.

**Results:**

① The median age of 282 patients was 46（15–66）years, with 191 males and 91 females. Among them, 118（42％）and 164（58％）had MDS-EB1 and MDS-EB2, respectively. ② Among the 282 patients, 256（90.8％）achieved hematopoietic reconstruction after transplantation, with 11（3.9％）and 15（5.3％）having primary and secondary implantation dysfunctions, respectively. The cumulative incidence of acute graft-versus-host disease（GVHD）100 days post-transplantation was（42.6±3.0）％, and the cumulative incidence of grade Ⅱ–Ⅳ acute GVHD was（33.0±2.8）％. The cumulative incidence of chronic GVHD 1 year post-transplantation was（31.0±2.9）％. Post-transplantation, 128（45.4％）, 63（22.3％）, 35（12.4％）, and 17 patients（6.0％）developed cytomegalovirus infection, bacteremia, pulmonary fungal infection, and Epstein-Barr virus infection. ③ The median follow-up time post-transplantation was 22.1（19.2–24.7）months, and the 3-year overall survival（OS）and disease-free survival（DFS）rates were 71.9％（95％*CI* 65.7％–78.6％）and 63.6％（95％*CI* 57.2％–70.7％）, respectively. The 3-year non-recurrent mortality rate（NRM）is 17.9％（95％*CI* 13.9％–22.9％）, and the 3-year cumulative recurrence rate（CIR）is 9.8％（95％*CI* 6.7％–13.7％）. The independent risk factors affecting OS post-transplantation include monocyte karyotype（*P*＝0.004, *HR*＝3.26, 95％*CI* 1.46–7.29）, hematopoietic stem cell transplantation complication index（HCI-CI）of ≥3 points（*P*<0.001, *HR*＝2.86, 95％*CI* 1.72–4.75）, and the occurrence of acute gastrointestinal GVHD of grade Ⅱ–Ⅳ（*P*<0.001, *HR*＝5.94, 95％*CI* 3.50–10.10）. ④ The 3-year OS and DFS rates in the MDS-IB1/IB2 group post-transplantation were better than those in the MDS-biTP53 group［OS: 72.0％（95％*CI* 63.4％–80.7％）*vs* 46.4％（95％*CI* 26.9％–80.1％）, *P*＝0.020; DFS: 67.4％（95％*CI* 60.3％–75.3％）*vs* 39.7％（95％*CI* 22.3％–70.8％）, *P*＝0.015］. The 3-year CIR was lower than that of the MDS-biTP53 group［7.3％（95％*CI* 4.3％–11.4％）*vs* 26.9％（95％*CI* 9.2％–48.5％）, *P*＝0.004］. The NRM at 3 years post-transplantation in the MDS-IB1/IB2, MDS-f, and MDS-biTP53 groups were 16.7％（95％*CI* 12.1％–22.1％）, 20.5％（95％*CI* 9.4％–34.6％）, and 26.3％（95％*CI* 9.1％–47.5％）, respectively（*P*＝0.690）.

**Conclusion:**

Allo-HSCT is an effective treatment for MDS-EB, with monomeric karyotype, HCI-CI, and grade Ⅱ–Ⅳ acute gastrointestinal GVHD as independent risk factors affecting the patient's OS. The WHO 2022 classification helps distinguish the efficacy of allo-HSCT in different subgroups of patients. Allo-HSCT can improve the poor prognosis of patients with MDS-f, but those with MDS-biTP53 have a higher risk of recurrence post-transplantation.

骨髓增生异常综合征（MDS）是一种高度异质性的血液系统肿瘤，MDS伴原始细胞增多（MDS-EB）是其中一种高危类型。该类型MDS对常规治疗反应差，具有较高的急性髓系白血病（AML）转化风险和较差的预后[1]–[2]。WHO 2016分类标准将MDS-EB根据原始细胞增多的程度分为MDS-EB1和MDS-EB2两个亚型[3]。WHO 2022分类标准重新命名了骨髓增生异常肿瘤（myelodysplastic neoplasm, MDS），并在分子学及病理学特征的基础上，将骨髓增生异常肿瘤伴原始细胞增多（MDS with increased blasts, MDS-IB）分为MDS-IB1、MDS-IB2及MDS伴纤维化（MDS with fibrosis, MDS-f）三个亚型，同时将伴双等位基因TP53突变的MDS（MDS with biallelic TP53 inactivation, MDS-biTP53）这一高危亚型重新单独归类为伴特定遗传学异常的MDS亚型之一，诊断优先级高于形态学定义的MDS，以有效识别更高危的患者[4]。异基因造血干细胞移植（allo-HSCT）可显著延长MDS患者的生存期，但如何识别高危患者并提高移植后生存率是目前亟待解决的问题。本研究回顾性分析在本中心接受allo-HSCT的282例MDS-EB患者的临床特征和生存结局、探讨影响患者生存的相关因素，并比较WHO 2022分类MDS-IB1/IB2、MDS-f、MDS-biTP53三组患者的预后。

## 病例与方法

一、研究对象

本研究对2006年10月至2022年12月在中国医学科学院血液病医院移植中心首次接受全相合同胞供者造血干细胞移植（MSD-HSCT）、全相合无关供者造血干细胞移植（MUD-HSCT）和单倍体造血干细胞移植（haplo-HSCT）的282例MDS-EB患者进行回顾性分析。纳入标准：①年龄≥14岁；②临床及随访资料完整。排除标准：①治疗相关或继发性MDS；②符合WHO 2022分类标准的AML伴NPM1患者。

二、治疗方法

所有患者均采用改良Bu/Cy清髓性预处理方案（DAC/Bu/Cy/Flu/Ara-C或Bu/Cy/Flu/Ara-C）。急性移植物抗宿主病（GVHD）预防采用环孢素A（CsA）或他克莫司（FK506）联合短疗程甲氨蝶呤（MTX）±霉酚酸酯（MMF）方案。MUD-HSCT和haplo-HSCT患者加用兔抗人胸腺细胞免疫球蛋白（rATG）2～2.5 mg·kg^−1^·d^−1^（−4 d～−1 d）。移植前应用膦甲酸钠或缬更昔洛韦等预防巨细胞病毒（CMV）感染，应用复方磺胺甲噁唑预防卡氏肺孢子菌感染，应用阿苯达唑清除肠道寄生虫等。GVHD的治疗一线首选方案为糖皮质激素，二线方案包括芦可替尼、钙调磷酸酶抑制剂（环孢素A、他克莫司、西罗莫司等）、间充质干细胞输注、抗CD25单抗等。

三、随访及研究终点

随访资料来自门诊或住院病历及电话随访，随访截止日期为2023年9月1日。主要终点为总生存（OS）时间，次要终点为无病生存（DFS）率、非复发死亡率（NRM）、累积复发率（CIR）。OS时间定义为从移植到死亡（任何原因）或末次随访之间的时间，DFS时间定义为从达到完全缓解（CR）到复发的时间，NRM定义为任何没有疾病复发的死亡。通过竞争风险法估计累积复发率（CIR）和NRM。

四、疗效标准及定义

MDS诊断参照《骨髓增生异常综合征中国诊断与治疗指南（2019年版）》[5]及WHO 2016分类标准[3]，MDS-IB1/IB2、MDS-f、MDS-biTP53的诊断参照WHO 2022分类标准[4]。移植前疗效评价参照IWG2006年修订疗效标准[6]，有治疗反应包括完全缓解（CR）、部分缓解（PR）、骨髓CR、稳定（SD）及血液学进步（HI），无治疗反应包括：失败、进展（PD）、HI后进展或复发。急性和慢性GVHD诊断和分级标准参照西雅图分级诊断标准[7]–[8]。

五、统计学处理

采用R 4.2.3软件进行统计学分析，计数资料以例数和构成比（％）的形式表示，计量资料采用“中位数（最小值～最大值）”表示，组间比较采用*t*检验或*χ*^2^检验。单因素生存分析采用Kaplan-Meier法，多因素生存分析采用Cox比例风险回归模型，以双侧*P*<0.05为差异有统计学意义。

## 结果

一、临床资料

282例MDS-EB患者中男191例，女91例，中位年龄46（15～66）岁，其中MDS-EB1患者118例（42％），MDS-EB2患者164例（58％）。按照WHO2022诊断标准重新分类：MDS-IB1 90例（31.0％）、MDS-IB2患者132例（45.5％）、MDS-f 41例（41.1％）、MDS-biTP53 19例（6.6％）。因临床特征及结局相似，本研究将MDS-IB1、MDS-IB2合并为一组。MDS-f组和MDS-biTP53组移植前铁蛋白水平高于MDS-IB1/IB2组（*P*＝0.002），MDS-biTP53组的单体核型（MK）患者占比及修订的国际预后评分系统（IPSS-R）细胞遗传学分层差/极差患者占比显著高于其他两组（*P*<0.001），MDS-IB1/IB2组患者骨髓原始细胞占比高于其他组（*P*＝0.048），详见表1。

**表1:** MDS-IB1/IB2、MDS-f、MDS-biTP53三组患者临床资料比较

指标	总体	WHO 2022诊断标准分组				
		MDS-IB1/IB2组（222例）	MDS-biTP53组（19例）	MDS-f组（41例）	统计量	*P*值
性别［例（%）］					*χ*^2^＝2.850	0.240
男	191（66.7）	145（65.0）	15（79.0）	31（76.0）		
女	91（32.3）	77（35.0）	4（21.0）	10（24.0）		
移植年龄［岁，*M*（范围）］	46（15~66）	46（15~64）	49（32~63）	44（15~66）	*χ*^2^＝2.910	0.230
诊断到移植时间［月，*M*（范围）］	3.9（0.5~64.8）	3.8（0.5~44.0）	3.9（0.6~13.5）	3.9（0.9~64.8）	*χ*^2^＝0.587	0.750
初诊血常规［*M*（范围）］						
HGB（g/L）	75（32~151）	75（32~151）	79（50~108）	73（43~125）	*t*＝1.340	0.510
ANC（×10^9^/L）	0.86（0~19.14）	0.84（0~19.14）	0.96（0~3.10）	0.95（0.07~5.40）	*t*＝1.970	0.370
PLT（×10^9^/L）	43（2~391）	44（2~306）	47（6~391）	32（6~360）	*t*＝2.645	0.270
初诊时骨髓原始细胞比例［%，*M*（范围）］	9（0~19.5）	9.5（1.0~17.5）	7.0（1.0~19.0）	7.5（0.0~19.5）	*t*＝6.053	0.048
单体核型［例（%）］	21（7.4）	12（5.4）	7（37.0）	2（4.9）		<0.001
伴TP53基因/细胞遗传学17p异常［例（%）］	31（11.0）	12（5.4）	14（74.0）	5（12.0）	*χ*^2^＝140.150	<0.001
IPSS-R危险度分层［例（%）］					*χ*^2^＝8.295	0.217
极低危+低危	7（2.5）	5（2.3）	0（0）	2（4.9）		
中危	38（13.5）	29（13.0）	2（11.0）	7（17.1）		
高危	117（41.5）	99（45.0）	4（21.0）	14（34.1）		
极高危	120（42.5）	89（40.0）	13（68.0）	18（43.9）		
IPSS-R细胞遗传学分层［例（%）］					*χ*^2^＝45.537	<0.001
好+极好	129（45.7）	111（50.0）	3（15.8）	15（36.6）		
中等	93（33.0）	78（35.1）	5（26.3）	10（24.4）		
差	28（9.9）	16（7.2）	2（10.5）	10（24.4）		
极差	30（10.6）	15（6.8）	9（47.4）	6（14.6）		
无法评估	2（0.7）	2（0.9）	0	0		
IPSS-M危险度分层［例（%）］					*χ*^2^＝14.644	0.066
极低危+低危	6（2.1）	6（2.7）	0（0）	0（0）		
中低危	12（4.3）	10（4.5）	0（0）	2（4.9）		
中高危	29（10.3）	25（11.3）	0（0）	4（9.8）		
高危	78（27.7）	66（29.7）	2（10.5）	10（24.4）		
极高危	123（43.6）	90（40.5）	17（89.5）	16（39.0）		
无法评估	34（12.0）	25（11.3）	0（0）	9（21.9）		
移植前治疗［例（%）］					*χ*^2^＝4.927	0.553
支持治疗	142（50.4）	115（51.8）	7（36.8）	20（48.9）		
HMA	65（23.0）	47（21.2）	7（36.8）	11（26.8）		
HMA联合化疗	60（21.3）	48（21.6）	5（26.3）	7（17.1）		
化疗	15（5.3）	12（5.4）	0（0）	3（7.3）		
移植前疗效评价［例（%）］					*χ*^2^＝8.648	0.071
支持治疗	138（48.9）	113（50.9）	6（31.6）	19（46.3）		
无治疗反应	74（26.2）	58（26.1）	9（47.4）	7（17.0）		
有治疗反应	70（24.8）	51（23.0）	4（21.0）	15（36.6）		
移植前铁蛋白［µg/L，*M*（范围）］	727（178~7 152）	667（178~6 464）	974（257~3 444）	1226（199~7 152）	*t*＝0.187	0.002
HCT-CI ≥ 3分［例（%）］	41（14.5）	31（14.0）	4（21.1）	6（14.6）	*χ*^2^＝33.122	0.700
供者类型［例（%）］					*χ*^2^＝3.092	0.700
MSD	114（40.4）	89（40.0）	6（31.6）	19（46.3）		
haplo	154（54.6）	120（54.1）	13（68.4）	21（51.2）		
MUD	14（5.0）	13（5.9）	0（0）	1（2.4）		
预处理方案含DAC［例（%）］					*χ*^2^＝4.482	0.110
否	77（27.3）	60（27.0）	2（10.5）	15（36.6）		
是	205（72.7）	162（73.0）	17（89.5）	26（63.4）		
MNC回输量［×10^8^/kg，*M*（范围）］	10.6（2.0~27.7）	10.9（2.0~27.7）	10.2（3.0~16.6）	10.0（4.8~23.4）	*t*＝2.336	0.311
CD34^+^细胞回输量［×10^6^/kg，*M*（范围）］	3.16（0.26~12.00）	3.00（1.20~8.04）	3.51（1.60~9.98）	3.54（1.42~12.00）	*t*＝1.101	0.577
中性粒细胞植入时间［d，*M*（范围）］	13（8~24）	13（8~22）	14（10~24）	14（10~23）	*t*＝2.977	0.200
血小板植入时间［d，*M*（范围）］	17（10~407）	17（11~407）	18（10~60）	19（10~81）	*t*＝3.710	0.200

**注** MDS-IB1/IB2：骨髓增生异常肿瘤伴原始细胞增多1型/2型；MDS-f：MDS伴纤维化；MDS-biTP53：伴双等位基因TP53突变的MDS；IPSS-R：修订的国际预后评分系统；IPSS-M：国际分子预后评分系统；HMA：去甲基化药物；HCT-CI：造血干细胞移植合并症指数；MSD：HLA全相合同胞供者；haplo：单倍体供者；MUD：HLA全相合无关供者；DAC：地西他滨；MNC：单个核细胞

二、造血重建、GVHD及移植后感染的发生情况

282例患者中有256例（90.8％）移植后获得造血重建，原发植入功能不良11例（3.9％），继发植入功能不良15例（5.3％）。移植后100 d急性GVHD累积发生率为（42.6±3.0）％，中位发生时间为移植后36 d，Ⅱ～Ⅳ度急性GVHD累积发生率为（33.0±2.8）％，Ⅱ～Ⅳ级肠道急性GVHD累积发生率为（16.7±2.2）％。107例患者发生慢性GVHD，中位发生时间为移植后210 d，移植后1年慢性GVHD累积发生率为（31.0±2.9）％，移植后2年慢性GVHD累积发生率为（41.4±3.3）％。128例（45.4％）患者在移植后发生CMV感染，其中26例（9.2％）为多部位CMV感染。63例（22.3％）患者移植后发生菌血症。35例（12.4％）患者合并肺部真菌感染，17例（6.0％）患者移植后合并EBV感染。

三、生存及预后危险因素分析

282例MDS-EB患者移植后中位随访时间为22.1（19.2～24.7）个月，3年OS率为71.9％（95％*CI* 65.7％～78.6％），3年DFS率为63.6％（95％*CI* 57.2％～70.7％），3年NRM为17.9％（95％*CI* 13.9％～22.9％），3年CIR为9.8％（95％*CI* 6.7％～13.7％）。

将患者年龄、HCT-CI、诊断时血常规、危险度分层、移植前治疗、移植前疾病状态及移植后感染、GVHD等生存相关因素纳入单因素分析，将单因素分析中*P*<0.1的因素纳入多因素分析，以双侧*P*<0.05为差异有统计学意义。结果显示：MK（*P*＝0.004，*HR*＝3.26，95％ *CI* 1.46～7.29）、HCI-CI≥ 3分（*P*<0.001，*HR*＝2.86，95％*CI* 1.72～4.75）、发生Ⅱ～Ⅳ度肠道急性GVHD（*P*<0.001，*HR*＝5.94，95％ *CI* 3.50～10.10）是影响OS的独立危险因素（表2）。

**表2:** 影响282例骨髓增生异常综合征伴原始细胞增多患者异基因造血干细胞移植后总生存的预后因素分析

因素	单因素分析			多因素分析		
	*HR*	95%*CI*	*P*值	*HR*	95%*CI*	*P*值
HCI-CI≥3分	3.02	1.86~4.93	<0.001	2.86	1.72~4.75	<0.001
单体核型	4.93	2.77~8.78	<0.001	3.26	1.46~7.29	0.004
Ⅱ~Ⅳ度肠道急性GVHD	5.77	3.60~9.26	<0.001	5.94	3.50~10.10	<0.001
IPSS-R细胞遗传学分级	1.50	1.21~1.86	<0.001	0.98	0.73~1.31	0.900
多部位CMV感染	2.88	1.60~5.17	<0.001	1.14	0.58~2.25	0.700
移植前血清铁蛋白>1800 µg/L	1.87	1.08~3.22	0.025	1.23	0.69~2.22	0.500

**注** HCT-CI：造血干细胞移植合并症指数；GVHD：移植物抗宿主病；IPSS-R：修订的国际预后积分系统；CMV：巨细胞病毒

四、WHO2022分类不同亚型移植后生存比较

MDS-IB1/IB2组、MDS-f组、MDS-biTP53组移植后3年OS率分别为72.0％（95％*CI* 63.4％～80.7％）、71.4％（95％*CI* 58.3％～87.5％）、46.4％（95％*CI* 26.9％～80.1％）。MDS-IB1/IB2组优于MDS-biTP53组（*P*＝0.020）（图1A），与MDS-f组比较差异无统计学意义（*P*＝0.640），MDS-biTP53组与MDS-f组比较差异亦无统计学意义（*P*＝0.060）。

**图1:**
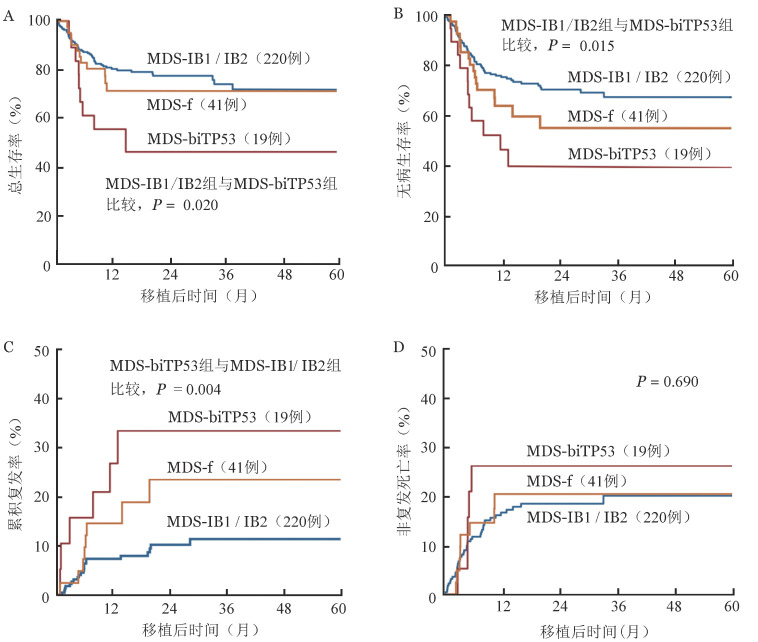
282例骨髓增生异常综合征伴原始细胞增多（MDS-EB）患者根据WHO 2022再分类后不同亚型的总生存曲线（A）、无病生存曲线（B）、复发曲线（C）和非复发死亡曲线（D） **注** MDS-IB1/IB2：骨髓增生异常肿瘤伴原始细胞增多1型/2型；MDS-f：骨髓增生异常肿瘤伴纤维化；MDS-biTP53：伴双等位基因TP53突变的骨髓增生异常肿瘤

MDS-IB1/IB2组、MDS-f组、MDS-biTP53组移植后3年DFS率分别为67.4％（95％*CI* 60.3％～75.3％）、55.0％（95％*CI* 40.0％～75.6％）、39.7％（95％*CI* 22.3％～70.8％）（*P*＝0.017），MDS-IB1/IB2组优于MDS-biTP53组（*P*＝0.015），MDS-IB1/IB2组与MDS-f组、MDS-f组与MDS-biTP53组比较差异均无统计学意义（*P*＝0.125，*P*＝0.270）（图1B）。

MDS-IB1/IB2组、MDS-f组、MDS-biTP53组移植后3年CIR分别为7.3％（95％*CI* 4.3％～11.4％）、14.6％（95％*CI* 5.8％～27.2％）、26.9％（95％*CI* 9.2％～48.5％），MDS-IB1/IB2组低于MDS-biTP53组（*P*＝0.004），MDS-biTP53组与MDS-f组、MDS-IB1/IB2组与MDS-f组比较差异均无统计学意义（*P*＝0.250，*P*＝0.120）（图1C）。

MDS-IB1/IB2组、MDS-f组、MDS-biTP53组移植后3年NRM分别为16.7％（95％*CI* 12.1％～22.1％）、20.5％（95％*CI* 9.4％～34.6％）、26.3％（95％*CI* 9.1％～47.5％），差异无统计学意义（*P*＝0.690）（图1D）。MDS-IB1/IB2组与MDS-f组、MDS-IB1/IB2组与MDS-biTP53组、MDS-f组与MDS-biTP53组比较，差异均无统计学意义（*P*＝0.554，*P*＝0.290，*P*＝0.616）。

## 讨论

allo-HSCT是治疗高危/极高危MDS患者的有效手段，与非移植患者相比，allo-HSCT能够显著改善晚期或高危MDS患者的生存[1]。多项大型国际合作组研究和单中心研究显示，MDS患者接受allo-HSCT治疗后，3年OS率为30％～63.6％，DFS率为16％～58.5％，移植相关死亡率（TRM）为10％～50％[9]–[13]。移植时机对MDS患者的预后也有影响，诊断后早期移植能够使IPSS评分中危-2或高危患者获得更长的生存期，而延迟移植则有利于提高IPSS评分低危或中危-1的患者的生存率[14]–[15]。本研究中282例MDS-EB患者按照IPSS-R及IPSS-M危险度评分大多属于高危/极高危组，移植后3年OS率为71.8％，DFS率63.8％，诊断到移植中位时间3.8个月，总体生存情况相对较好，这可能与本研究纳入的患者相对年轻、诊断至移植的时间相对较短，以及采用清髓性预处理方案有关。这些结果提示MDS-EB患者能够显著受益于清髓性预处理allo-HSCT。

骨髓纤维化（BMF）是MDS的一种不良预后因素，与多系发育不良、严重的血小板减少、高比例的克隆性核型异常以及外周血中原始细胞增多相关[16]–[18]。研究证实，MDS-f中位生存时间仅为10个月，明显短于MDS-IB其他亚组[19]。allo-HSCT能否克服骨髓纤维化的不利因素存在争议。一些研究认为骨髓纤维化可能与较低的移植后生存率、较高的复发率和NRM有关[20]–[22]。同时也有研究认为，在MDS患者中，只有严重的骨髓纤维化会影响allo-HSCT后生存率，而轻度或中度骨髓纤维化患者的预后与未发生骨髓纤维化患者相当[22]。本组病例中，MDS-f组allo-HSCT后3年OS率为71.4％（95％*CI* 58.3％～87.5％），DFS率为55.0％（95％*CI* 40.0％～75.6％），NRM为20.5％（95％*CI* 9.4％～34.6％），与MDS-IB1/IB2组比较差异无统计学意义，提示allo-HSCT能够改善MDS-f的不良预后，是MDS-f患者一种值得尽早选择的有效治疗方法。

BiTP53突变确定了一个独立于IPSS-R和共突变模式的极高风险亚型，具有复杂的细胞遗传学、较少的共突变、快速的疾病进展和耐药性等临床特征[23]–[24]。一些研究表明TP53突变所驱动的显性负性效应是导致不良预后的重要原因[25]。Grob等[24]研究发现，因分子特性和生存结局的相似性，伴TP53突变AML/MDS-EB应被认为是单一分子疾病实体。allo-HSCT是唯一可长期治愈TP53突变患者的治疗方法，但OS率仅为10％～20％[26]。移植后的早期复发是TP53突变患者allo-HSCT后死亡的主要原因。本研究中19例MDS-biTP53同时伴有原始细胞增高，移植后复发率较高，3年OS率虽显著低于MDS-IB患者，但DFS率仍可以达到39.7％，提示allo-HSCT仍为部分MDS-biTP53患者提供了长期生存的可能。优化治疗方案如在预处理方案中增加维奈克拉或移植后应用阿扎胞苷进行维持治疗的临床试验取得了一些有前景的效果[27]–[28]，需要大系列的前瞻性随机对照研究来确定MDS-biTP53患者最佳移植治疗方案。

本研究对影响患者生存的因素进一步统计分析，结果显示：MK、HCT-CI、Ⅱ～Ⅳ度肠道急性GVHD是影响MDS-EB患者生存的独立危险因素。MK是allo-HSCT预后不良的一个重要危险因素，研究发现MK与TP53突变存在显著的关联性[29]–[30]。同时也有研究发现并不是MK本身，而是与MK相关的额外细胞遗传学异常如复杂核型（CK）与移植后较高的CIR和较短的OS有关[31]。HCT-CI也是影响生存的独立危险因素，通过HCT-CI评估的机体功能状态是比实际年龄更重要的移植预后因素，对于预测NRM至关重要[32]。本研究结果与其他研究结论一致。移植后并发症是导致MDS移植后死亡的主要原因，本研究结果显示Ⅱ～Ⅳ度肠道急性GVHD是影响移植后生存的独立危险因素。高达50％的急性肠道急性GVHD患者为糖皮质激素抵抗，需要启动二线治疗[33]–[35]。目前已有多个新药正在开展临床研究，同时在开发利用生物标记物早期预测肠道急性GVHD的发生和治疗反应[36]–[37]。

本研究结果显示WHO2022分类有助于识别更高危的MDS患者，但因再分类后亚组样本量差异较大可能使结果产生一定的偏移，同时本研究纳入的MDS-biTP53患者均伴有原始细胞增高，对于不伴原始细胞增高的MDS-biTP53患者后期需要纳入更多样本进行额外的研究。

综上，本研究结果显示：MDS-EB患者显著获益于早期清髓性预处理方案allo-HSCT；MK、HCT-CI、Ⅱ～Ⅳ度肠道急性GVHD是影响MDS-EB患者生存的独立危险因素；allo-HSCT能够改善MDS-f的不良预后，MDS-biTP53患者移植后仍然存在较高的复发风险，需要继续探索新的治疗方案。

## References

[1] Garcia-Manero G (2023). Myelodysplastic syndromes: 2023 update on diagnosis, risk-stratification, and management[J]. Am J Hematol.

[2] Cazzola M (2011). Risk assessment in myelodysplastic syndromes and myelodysplastic/myeloproliferative neoplasms[J]. Haematologica.

[3] Arber DA, Orazi A, Hasserjian R (2016). The 2016 revision to the World Health Organization classification of myeloid neoplasms and acute leukemia[J]. Blood.

[4] Khoury JD, Solary E, Abla O (2022). The 5th edition of the World Health Organization Classification of Haematolymphoid Tumours: Myeloid and Histiocytic/Dendritic Neoplasms[J]. Leukemia.

[5] 中华医学会血液学分会 (2019). 骨髓增生异常综合征中国诊断与治疗指南(2019年版)[J]. 中华血液学杂志.

[6] Cheson BD, Greenberg PL, Bennett JM (2006). Clinical application and proposal for modification of the International Working Group (IWG) response criteria in myelodysplasia[J]. Blood.

[7] Przepiorka D, Weisdorf D, Martin P (1995). 1994 Consensus Conference on Acute GVHD Grading[J]. Bone Marrow Transplant.

[8] Jagasia MH, Greinix HT, Arora M (2015). National Institutes of Health Consensus Development Project on Criteria for Clinical Trials in Chronic Graft-versus-Host Disease: I. The 2014 Diagnosis and Staging Working Group report[J]. Biol Blood Marrow Transplant.

[9] Baron F, Zachée P, Maertens J (2015). Non-myeloablative allogeneic hematopoietic cell transplantation following fludarabine plus 2 Gy TBI or ATG plus 8 Gy TLI: a phase II randomized study from the Belgian Hematological Society[J]. J Hematol Oncol.

[10] Benjamin J, Chhabra S, Kohrt HE (2014). Total lymphoid irradiation-antithymocyte globulin conditioning and allogeneic transplantation for patients with myelodysplastic syndromes and myeloproliferative neoplasms[J]. Biol Blood Marrow Transplant.

[11] Chang C, Storer BE, Scott BL (2007). Hematopoietic cell transplantation in patients with myelodysplastic syndrome or acute myeloid leukemia arising from myelodysplastic syndrome: similar outcomes in patients with de novo disease and disease following prior therapy or antecedent hematologic disorders[J]. Blood.

[12] De Witte T, Hermans J, Vossen J (2000). Haematopoietic stem cell transplantation for patients with myelo-dysplastic syndromes and secondary acute myeloid leukaemias: a report on behalf of the Chronic Leukaemia Working Party of the European Group for Blood and Marrow Transplantation (EBMT)[J]. Br J Haematol.

[13] Kim YJ, Jung SH, Hur EH (2018). TP53 mutation in allogeneic hematopoietic cell transplantation for de novo myelodysplastic syndrome[J]. Leuk Res.

[14] Cutler CS, Lee SJ, Greenberg P (2004). A decision analysis of allogeneic bone marrow transplantation for the myelodysplastic syndromes: delayed transplantation for low-risk myelodysplasia is associated with improved outcome[J]. Blood.

[15] Koreth J, Pidala J, Perez WS (2013). Role of reduced-intensity conditioning allogeneic hematopoietic stem-cell transplantation in older patients with de novo myelodysplastic syndromes: an international collaborative decision analysis[J]. J Clin Oncol.

[16] Melody M, Al Ali N, Zhang L (2020). Decoding bone marrow fibrosis in myelodysplastic syndromes[J]. Clin Lymphoma Myeloma Leuk.

[17] Buesche G, Teoman H, Wilczak W (2008). Marrow fibrosis predicts early fatal marrow failure in patients with myelodysplastic syndromes[J]. Leukemia.

[18] Zhao YS, Guo J, Zhao SD (2022). Bone marrow fibrosis at diagnosis and during the course of disease is associated with tp53 mutations and adverse prognosis in primary myelodysplastic syndrome[J]. Cancers (Basel).

[19] Zhang Y, Wu J, Qin T (2022). Comparison of the revised 4th (2016) and 5th (2022) editions of the World Health Organization classification of myelodysplastic neoplasms[J]. Leukemia.

[20] Rajantie J, Sale GE, Deeg HJ (1986). Adverse effect of severe marrow fibrosis on hematologic recovery after chemoradiotherapy and allogeneic bone marrow transplantation[J]. Blood.

[21] Wang N, Xu H, Li Q (2020). Patients of myelodysplastic syndrome with mild/moderate myelofibrosis and a monosomal karyotype are independently associated with an adverse prognosis: long-term follow-up data[J]. Cancer Manag Res.

[22] Kröger N, Zabelina T, Van Biezen A (2011). Allogeneic stem cell transplantation for myelodysplastic syndromes with bone marrow fibrosis[J]. Haematologica.

[23] Bernard E, Nannya Y, Hasserjian RP (2020). Implications of TP53 allelic state for genome stability, clinical presentation and outcomes in myelodysplastic syndromes[J]. Nat Med.

[24] Grob T, Al Hinai ASA, Sanders MA (2022). Molecular characterization of mutant TP53 acute myeloid leukemia and high-risk myelodysplastic syndrome[J]. Blood.

[25] Boettcher S, Miller PG, Sharma R (2019). A dominant-negative effect drives selection of TP53 missense mutations in myeloid malignancies[J]. Science.

[26] Daver NG, Maiti A, Kadia TM (2022). TP53-mutated myelodysplastic syndrome and acute myeloid leukemia: biology, current therapy, and future directions[J]. Cancer Discov.

[27] Garcia JS, Kim HT, Murdock HM (2021). Adding venetoclax to fludarabine/busulfan RIC transplant for high-risk MDS and AML is feasible, safe, and active[J]. Blood Adv.

[28] Oran B, De Lima M, Garcia-Manero G (2020). A phase 3 randomized study of 5-azacitidine maintenance vs observation after transplant in high-risk AML and MDS patients[J]. Blood Adv.

[29] Gauthier J, Damaj G, Langlois C (2015). Contribution of revised international prognostic scoring system cytogenetics to predict outcome after allogeneic stem cell transplantation for myelodysplastic syndromes: a study from the French Society of Bone Marrow Transplantation and Cellular Therapy[J]. Transplantation.

[30] Tefferi A, Idossa D, Lasho TL (2017). Mutations and karyotype in myelodysplastic syndromes: TP53 clusters with monosomal karyotype, RUNX1 with trisomy 21, and SF3B1 with inv(3)(q21q26.2) and del(11q)[J]. Blood Cancer J.

[31] Kelaidi C, Tzannou I, Baltadakis I (2014). Specific abnormalities versus number of abnormalities and cytogenetic scoring systems for outcome prediction after allogeneic hematopoietic SCT for myelodysplastic syndromes[J]. Bone Marrow Transplant.

[32] Atallah E, Logan B, Chen M (2020). Comparison of patient age groups in transplantation for myelodysplastic syndrome: the medicare coverage with evidence development study[J]. JAMA Oncol.

[33] Naymagon S, Naymagon L, Wong SY (2017). Acute graft-versus-host disease of the gut: considerations for the gastroenterologist [J]. Nat Rev Gastroenterol Hepatol.

[34] Ferrara JL, Levine JE, Reddy P (2009). Graft-versus-host disease[J]. Lancet.

[35] Deeg HJ (2007). How I treat refractory acute GVHD[J]. Blood.

[36] Patel DA, Crain M, Pusic I (2023). Acute graft-versus-host disease: an update on new treatment options[J]. Drugs.

[37] Choe H, Ferrara JLM (2021). New therapeutic targets and biomarkers for acute graft-versus-host disease (GVHD)[J]. Expert Opin Ther Targets.

